# Deconvoluting Patterson

**DOI:** 10.1107/S1600576725006569

**Published:** 2025-08-13

**Authors:** Bernhard Rupp

**Affiliations:** a Department of General, Inorganic and Theoretical Chemistry University of Innsbruck Innrain 80-82 Innsbruck6020 Austria; b k.-k. Hofkristallamt, San Diego, CA92084, USA; Instituto Andaluz de Ciencias de la Tierra, Granada, Spain

**Keywords:** Patterson function, autocorrelation, graphical animation, 1D electron density plots

## Abstract

The Patterson function, *P*(**u**), is useful in many crystallographic applications, including experimental phasing, molecular replacement and the exploration of non-crystallographic symmetries. *P*(**u**) is defined as the autocorrelation of the electron density. Autocorrelation is often an unfamiliar concept for students of biological or biomedical fields of study. I present a freely available animated *PowerPoint* slide deck featuring 1D electron density plots and the corresponding autocorrelation resulting in the Patterson function that is readily interpretable as an interatomic distance map. The animation makes it easy to grasp the concept of autocorrelation in an intuitive manner.

## Abstract 2

Implemented in many crystallography programs, the Patterson function can be challenging to explain to non-mathematically trained students. I provide a customizable animated *PowerPoint* slide deck to help students to intuitively grasp the concept of autocorrelation and its interpretation.

## 1. Background

Educators teaching the fundamentals of biomolecular crystallography to students with no specific mathematical background need to explain underlying concepts in a way that they can also be grasped intuitively by those focused on biological or biomedical curricula. Such students represent the majority of candidates in most biomolecular crystallography courses, and throwing unfamiliar and complex mathematical formulas at them with little context is seldom productive. The *PowerPoint* slide deck described in this manuscript illustrates the generation of the Patterson function through an intuitive and easy-to-understand animation.

The Patterson function *P*(**u**), named after Arthur Lindo Patterson (1902–1966), a pioneer in early X-ray crystallography (Patterson, 1934 ▸; Marsh & Shoemaker, 1967 ▸), works mostly underappreciated in the background, deeply buried in the code of crystallographic programs. Mathematically, *P*(**u**) is the autocorrelation of the electron density ρ(**r**), which explains absolutely nothing about its practical use and value in crystallography. A helpful discussion of *P*(**u**) should address the basic motivation of students: what is it good for?

## 2. No phase: a problem

We assume that, at this point of any biomolecular X-ray crystallography curriculum, students are aware that the measured intensities, 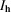, of reflections with indices **h** are proportional to the squared magnitudes or amplitudes, 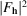, of the associated complex structure factors, 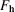, and that these scalar amplitudes alone are insufficient for the reconstruction of the electron density via inverse Fourier transformation (FT). The phase terms 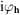 as the dominating second Fourier coefficients are also required and remain initially unknown. Almost every lecturer of macromolecular crystallography will likely have already shown a visualization of the phase problem as exemplified in an introductory slide of the *PowerPoint* deck.

Atomic positions determine phase (Blundell & Johnson, 1976 ▸; Rupp, 2009 ▸; Giacovazzo *et al.*, 2011 ▸) and, as a consequence, almost all macromolecular phasing techniques require atomic positions to recover phase information. As shall be demonstrated, *P*(**u**) allows us to recover atomic position information and, therefore, the Patterson function should be useful for recovering phase information. The question is how.

## 3. Autocorrelation in action

An autocorrelation function such as *P*(**u**) is the result of superpositions of a function with itself. In our case, the function to be superimposed and giving rise to the ‘overlap’ function *P*(**u**) is electron density ρ(**r**). For 1D electron density, where **r** and **u** become scalar, we can write down the autocorrelation integral as
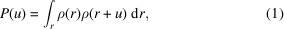
which instantaneously leads to a tune-out of most of the audience. To preserve the attention of the listeners, we should visualize and semiquantitatively analyze the result of the electron density autocorrelation in a more accessible manner.

**Figure 1:**
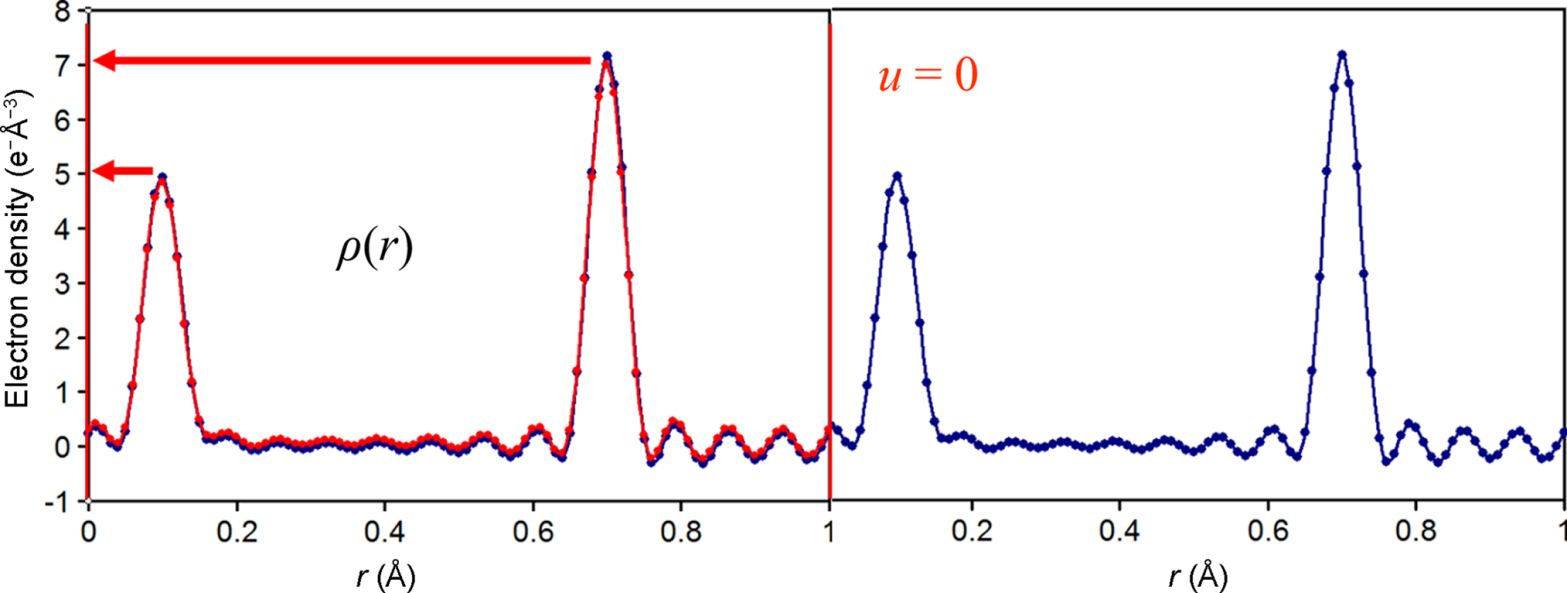
Snapshot at the start of the animation. The periodic electron density of two adjacent unit cells of a 1D structure is shown, superimposed with a copy of itself (in red) at shift *u* = 0.

We use interactively generated 1D electron density ρ(*r*) in a *PowerPoint* animation (*cf*. *Data availability*). This electron density function has peaks at *r* = 0.1 and 0.7 representing a 1D structure of two atoms, say C and O, and some noise from Fourier ripples. Fig. 1 ▸ shows a snapshot of the provided PowerPoint animation.

**Figure 2:**
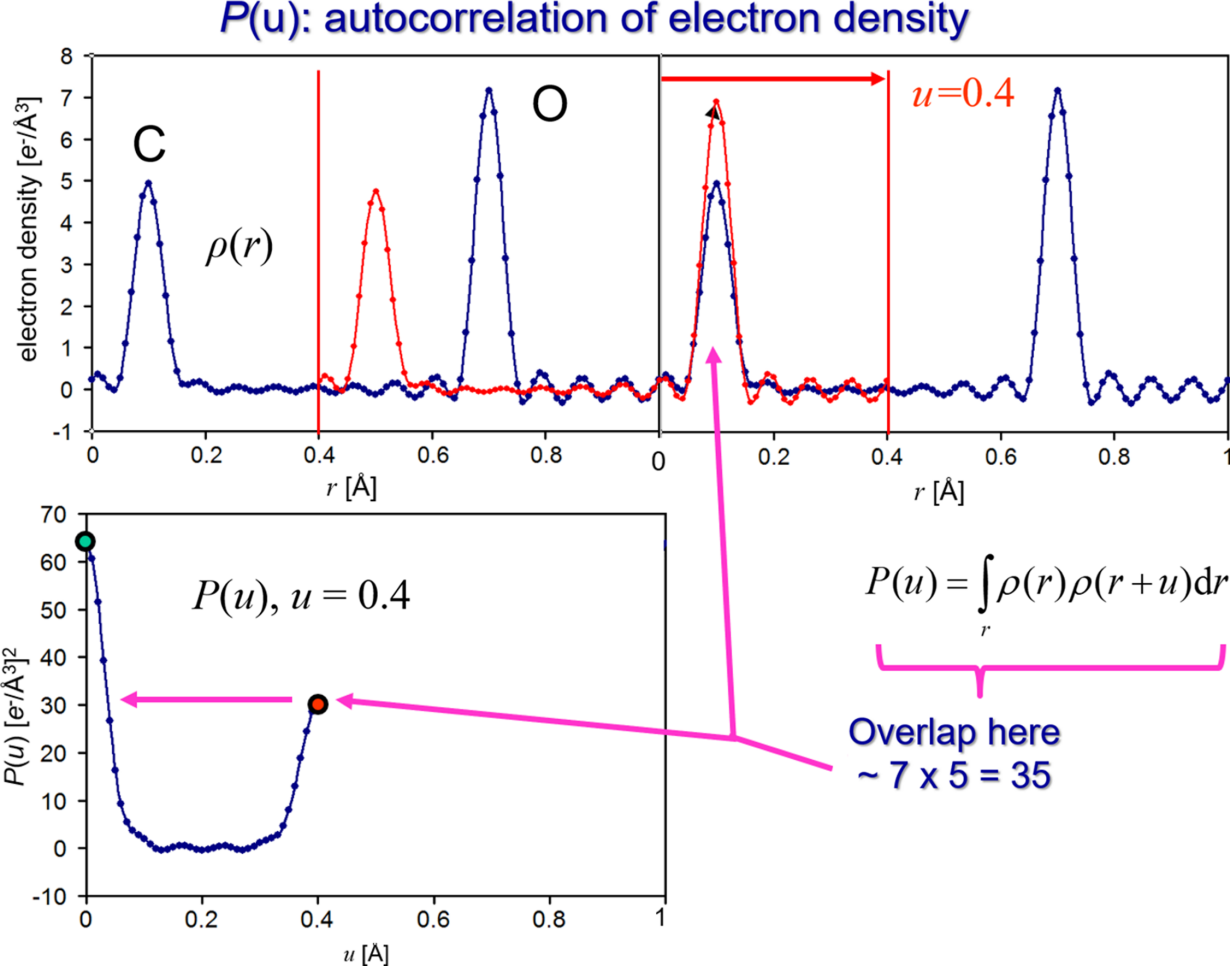
Snapshot of the animation at *u* = 0.4. The snapshot of progression of the superposition at *u* = 0.4 shows the appearance of a new peak, and the value of the autocorrelation integral *P*(0.4) can be approximated by the product of the peak heights (red dot). The value at *u* = 0 is indicated in green.

To determine the autocorrelation function *P*(*u*), we graphically shift the electron density along the *r* axis by a value of *u*, thus superimposing ρ(*r*) on itself. For *u* = 0 (Fig. 1 ▸), the integrand in the autocorrelation integral (1) reduces to a multiplication of the function with itself. We can thus approximate the value of the autoconvolution integration equation (1) by multiplying the peak heights of our electron density function in Fig. 1 ▸. We can read the peak heights off the electron density axis and for the initial overlap of the unshifted function (*u* = 0), the origin peak, we obtain *P*(0) ≃ (7 × 7) + (5 × 5) = 74. The first data point (green) at *u* = 0 in the *P*(*u*) panel of Fig. 2 ▸ provides the exact integration value plotted in the *P*(*u*) graphs and is close to our approximation.

We continue to shift the electron density graph over itself until we find the next Patterson peak at *u* = 0.4. We can estimate the magnitude again by multiplication of the respective peak heights: 7 × 5 = 35. We observe reasonable agreement with the accurate *P*(0.4) peak height in Fig. 2 ▸ (red dot).

We continue the shift by incrementing *u* until we reach *u* = 1 and obtain the complete Patterson function. The *PowerPoint* deck animates the progression of the superposition for all values 0 ≤ *u* ≤ 1.

## 4. How does this help us?

**Figure 3:**
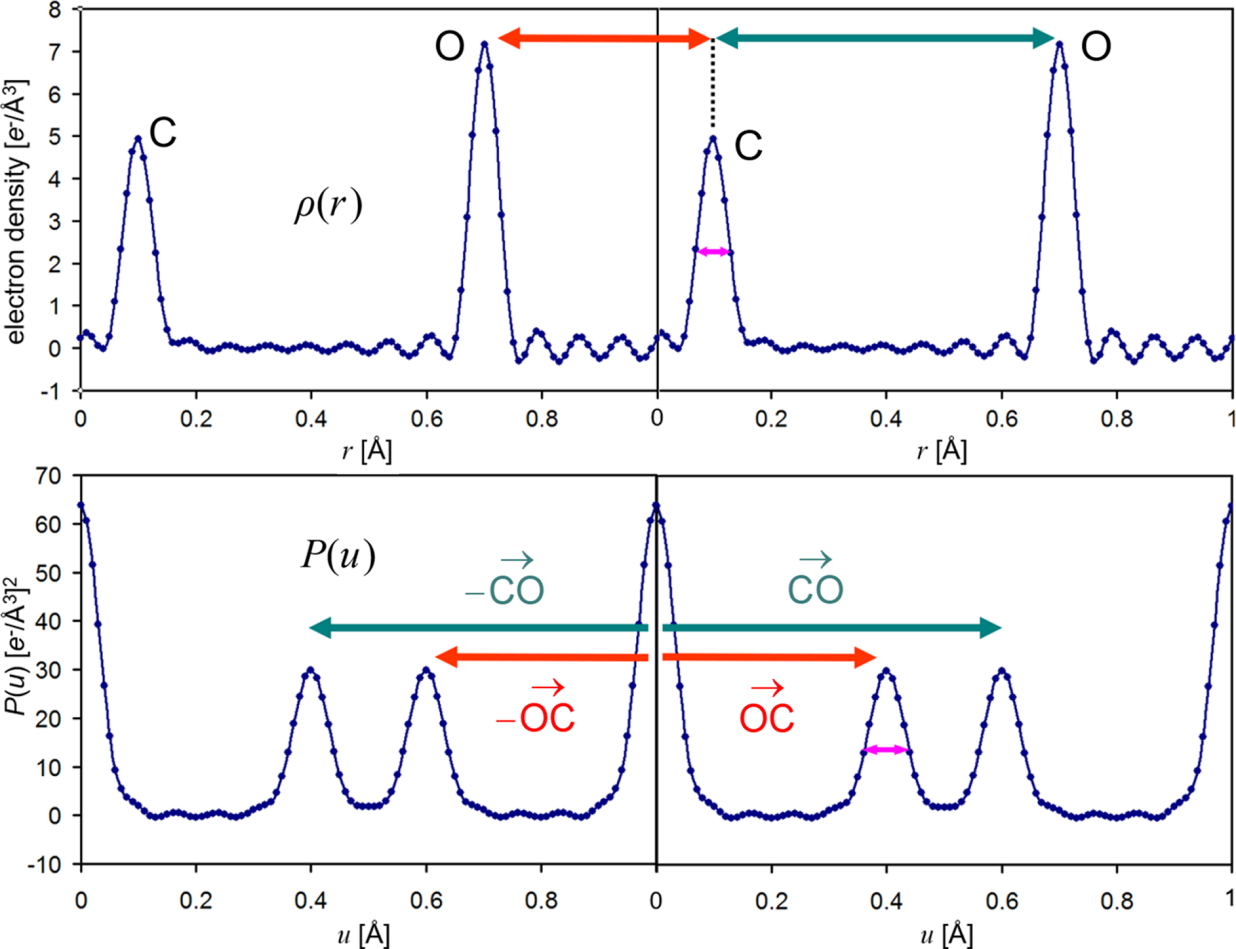
Comparing electron density with its autocorrelation function demonstrates that *P*(*u*) reveals the interatomic distances. The centrosymmetric nature of the interatomic distance vector map (Patterson map) in contrast to the electron density map becomes obvious. We also discover that, as a result of the convolution, the Patterson peaks are twice as broad as the electron density peaks (*cf*. purple arrows).

Now that we have been able to generate the overlap function graphically, we can compare the original electron density function ρ(*r*) with its autocorrelation *P*(*u*) (Fig. 3 ▸). From Fig. 3 ▸ we can extract several crucial relations. We realize that

(*a*) Patterson peaks appear at the tips of the interatomic distance vectors *r*(*i*) − *r*(*j*). The distance |*r*(*i*) − *r*(*j*)| between the C and O atom in the left density panel is 0.7 − 0.1 = 0.6, and between the O atom in the left density panel and the C in the consecutive right panel is 1.1 − 0.7 = 0.4.

(*b*) The function is always centrosymmetric, which in three dimensions implies a loss of handedness information, giving rise to the handedness ambiguity in the resulting substructure solutions.

(*c*) Given the *N* atoms or peaks in ρ(*r*), the function *P*(*u*) has *N*^2^ peaks in total, of which *N* are origin peaks. For our case with *N* = 2 we realize that the two origin peaks *P*(0) and *P*(1) are the largest peaks, and they are usually removed in Patterson peak searches. The remaining *N*^2^ − *N* = *N*(*N* − 1) Patterson peaks are cross-peaks (also two here). The *N*-squared increase of the number of peaks and the peaks being twice as broad compared with electron density peaks means that the Patterson map becomes crowded rapidly for large structures with many atoms. This suggests that *P*(*u*) should be useful for primarily two purposes:

(i) For *simple* situations such as small-molecule structure determination, or heavy or anomalous marker atom substructure determination, where the relatively few atom positions in a given (sub)structure can be calculated from the respective distance (Patterson) vectors, listed for example by Ward (1998 ▸). Knowledge of substructure atom positions is the basis of experimental substructure phasing techniques.

(ii) As a general correlation (overlap) function (Brünger, 1990 ▸) to determine the orientation and position of structurally similar molecules (which have similar interatomic distance maps) in unknown crystal structures, which is used in mol­ecular replacement (as in repositioning, not substitution) phasing (Rossmann, 2001 ▸).

In the situation of up to a few hundred atoms in biomol­ecular substructure determination, Patterson-seeded direct methods (Sheldrick, 1997 ▸) are a key computational ingredient in experimental substructure phasing techniques and are implemented for example in the *SHELXD* component of the *SHELX* program suite (Sheldrick, 2008 ▸).

## 5. An intense situation

At this point we know how *P*(*u*) relates to, and can be computed from, electron density. But because of the phase problem, we initially *do not know* the electron density. The necessary basic complex algebra for the derivation below is summarized in an optional slide in the *PowerPoint* deck.

The relation of the Patterson function to the measurable intensities can be derived using the Fourier convolution theorem (FCT). The FCT may have been encountered before in the crystallographic curriculum, at least visually/qualitatively, for instance in the convolution of the crystal lattice with the unit-cell content (Giacovazzo *et al.*, 2011 ▸; Rupp, 2009 ▸).

Briefly, in the generic convolution integral (using 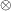 as the convolution operator) for two different functions *f*(**r**) and *g*(**r**),
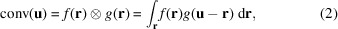
we replace *g*(**r**) with *g*(−**r**) which changes the second integrand to *g* (**r** + **u**) and the convolution into a correlation:
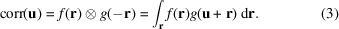
Next, we substitute ρ for both *g* and *f* (same function, thus ‘auto’ in correlation). The corresponding autocorrelation for ρ(**r**) then becomes
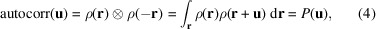
and in the 1D case, the convolution integral becomes our autocorrelation *P*(*u*) in equation (1).

Now we make use of the FCT stating that the FT of a convolution equals the product of the FTs of each operand:
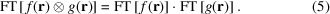
Substituting our autocorrelation function (4) into (5) we obtain
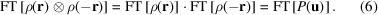
Equation (6) is exceptionally useful, because we already know (if not, a revision of the curriculum might be in order) that the FT of the real space electron density, 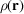, yields the set of complex structure factors, 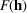, in reciprocal space:
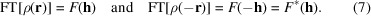
With 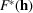 being the complex conjugate of 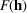, substituting the equivalencies (7) into (6) yields
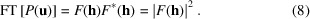
The above^1^ is certainly promising, because we have obtained the FT of *P*(**u**) now as a function of the square of the structure factor amplitudes 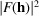, demonstrating that *P*(**u**) itself is also a function of the experimentally accessible, measured reflection intensities 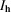. Inverting the FT, we obtain, in the form of the discrete Fourier summation,
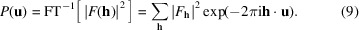
Applying Euler’s formula and exploiting the centrosymmetry of the reciprocal space in the summation eliminates the complex (Euler) sinus terms and we obtain the explicit real function useful for enumeration:
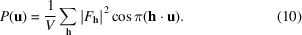
Now it is time to summarize the results.

## 6. Insights gained

The following summary provides a minimum of takeaways that should be retained and perhaps inspire useful test questions:

The Patterson function, defined as the autocorrelation of the electron density, can be computed from the experimentally accessible squared structure factor amplitudes, that is, *the Patterson function is intensity based*. The peaks in the Patterson function are *interatomic distance vectors*, from which the *atomic positions* of the atoms giving rise to the electron density can be computed. As *position determines phase*, the Patterson function is the basis for position based *macromolecular phasing techniques*.

Patterson seeding combined with direct methods is used to determine the position of up to a few hundred heavy or anomalous marker atom substructures of biomolecular structures. Knowledge of *substructure atom positions* is the basis of *experimental substructure phasing techniques*. Note that the Patterson function is *centrosymmetric*, which implies a loss of handedness information, giving rise to the *handedness ambiguity* in substructure solutions.

The Patterson function can be used as a general correlation function to determine the orientation and position of structurally similar molecules in unknown crystal structures, which is used in *molecular replacement phasing* (*e.g.* Rossmann, 2001 ▸).

A special form is the Patterson *self-rotation function*, which allows us to determine the *internal rotational symmetry* of a *non-crystallographic symmetry* assembly (motif) in a crystal structure from intensity data (Rossmann & Blow, 1962 ▸; McCoy & Read, 2025 ▸).

A *native Patterson map* can reveal a proper *non-crystallographic symmetry axis* (Wang & Janin, 1993 ▸) parallel or very nearly parallel to a crystallographic axis (Drenth, 2007 ▸; Rupp, 2009 ▸).

Time to dismiss class and elope for coffee.

## 7. Pedagogy

The explanation of the Patterson function as presented here fits comfortably into a 30 to 50 min session. A common challenge in teaching biomolecular courses is achieving a balance of intuitive and engaging delivery of complex (in both senses) mathematical concepts without abandoning formal rigor altogether. Often a blend of complementary presentations is necessary, as demonstrated in the example of the Patterson function: the superposition animation is quite illustrative and the relation of the Patterson function to interatomic distances is readily understood, while the proof that *P*(**u**) is intensity based does require some understanding of (or at least exposure to) FTs and basic complex algebra. Ultimately, how deeply a lecturer dives into each aspect depends on the subject as well as the audience, and instructors will have to tailor the mix of intuitive and rigorous content according to their students’ backgrounds.

## Data Availability

The *PowerPoint* animation can be downloaded from the password protected web page URL https://tinyurl.com/hofkristallamt. The password question should be easy to guess by anyone with a crystallographic background. The 1D structures, the corresponding electron density functions, and the associated Patterson functions and their graphs used in the production of the animated *PowerPoint* deck can be readily generated with the interactive web apps provided on my website https://tinyurl.com/Kristallapps. They can also be used for generating your own customized teaching material for the introduction of FT techniques. The figures in the downloadable animated *PowerPoint* deck and in the literature (Rupp, 2009 ▸) have been assembled using these web-generated images.

## Footnotes

1 Note the absence of the vector multiplication dot in the product *F*(**h**)(**h**) – complex numbers are not vectors: *cf.* sidebar 6–2 of Rupp (2009 ▸).
